# Thrombospondin Type 1 Domain-Containing 7A (THSD7A)-Associated Membranous Nephropathy Leading to Metastatic Neuroendocrine Carcinoma

**DOI:** 10.7759/cureus.35277

**Published:** 2023-02-21

**Authors:** Angel Juarez, Lidice Galindo, Aditya Ragunathan, Maryam Gondal

**Affiliations:** 1 Internal Medicine, Grand Strand Medical Center, Myrtle Beach, USA; 2 Nephrology, Grand Strand Medical Center, Myrtle Beach, USA

**Keywords:** low anion gap, thsd7a associated membranous glomerulopathy, paraneoplastic syndromes, small cell carcinomas, membranous nephropathy

## Abstract

Idiopathic membranous nephropathy also known as primary membranous nephropathy (PMN) is a common cause of nephrotic syndrome often seen in nondiabetic adults worldwide, rising as high as 40% in adults over the age of 60. Most PMN is mediated by antibodies to the M-type phospholipase A2 receptor (anti-PLA2R) in nearly 70%-80% of individuals. Thrombospondin type 1 domain-containing 7A (THSD7A) accounts for 1%-5% of individuals with PMN. In these individuals, malignancies have a varying incidence of 6%-25%. We present a case of idiopathic membranous nephropathy with anti-PLA2R negative and THSD7A positive with an underlying metastatic neuroendocrine carcinoma. Our goal is to highlight the importance of cancer screening in individuals with THSD7A-positive PMN. In addition, although nonspecific, a negative anion gap may be an indicator of an underlying malignancy.

## Introduction

Idiopathic membranous nephropathy (IMN) is the most common cause of primary nephrotic syndrome in adults. The classic presentation of membranous nephropathy (MN) is a patient with fatigue, malaise, and edema. Presentation is usually insidious in nature with one-third of patients at risk of progression to end-stage renal disease. The diagnosis of MN is based on renal biopsy. Anti-PLA2R was the first antigenic target to be recognized in IMN, discovered in 2009 [1-3]. THSD7A was described as a second autoantigen involved in IMN. The prevalence of THSD7A has a reported range of 1-5%. Among individuals with THSD7A-associated MN, studies have shown the development of malignancy within a median follow-up of three months from the time of initial diagnosis [3]. Due to this, physicians should be aware of the possible development of malignancy and emphasize age-appropriate cancer screening during routine outpatient visits.

## Case presentation

We present a 72-year-old female with a past medical history of hypothyroidism, tobacco use, and endometrial cancer status post hysterectomy at the age of 32 presenting with worsening bilateral lower extremity edema. The patient was at her baseline state of health up until three weeks prior to presentation. She had seen her primary physician for lower extremity edema and was started on furosemide with no major improvement. The patient noted weight gain at an estimated 40 lbs. In addition, she endorsed minimal dysphagia which was described as a “lump in her throat”. On further questioning, she reports excruciating neuropathic pain involving bilateral hands and feet for the past year. She also notes that she first noticed lower extremity edema two years prior to this presentation mainly localized to her bilateral ankles. Physical exam WAs remarkable for minimal wheezing with no noted crackles in bilateral lung exam, non-tender bilateral lower extremities, 3+ pitting edema, and minimal periorbital edema. No Jugular venous distention or abdominal distension was noted. The patient had a significant 60-pack-year history of tobacco use. The patient had a notable urinalysis one year prior with urine protein 200 mg/dL.

Initial blood work showed Hemoglobin 11.2 g/dL (normal values 11.6-15.4), white blood cell count 7.7 k/mm^3^ (normal values 3.7-10.1), hematocrit 33.9% (normal values 34.9%-44.1%), platelet count 220 k/mm^3^ (normal values 156-352), sodium 134 mmol/L (normal values 137-146), chloride 108 mmol/L, potassium 4.0 mmol (normal values 3.5-5.1), CO_2_ 34 mmol/L (normal values 22-32), BUN 32 mg/dL (normal values 7-20), creatinine 1.10 mg/dL (normal values 0.7-1.5), Est GFR 53 (normal values > 60), glucose 99 mg/dL (normal values 74-106), anion gap - 2 mEq/L (normal values 3.0-11.0), albumin 2.2 g/dL (normal 3.5-5.0), thyroid-stimulating hormone 3.42 uII/mL (normal values 0.465-4.68). Urine studies were notable for urine random total protein 700 mg/dL, urine creatinine 12 mg/dL, urine sodium 64 mmol/L, urine chloride 55 mmol/L, urine potassium 12.9 mmol/L, urine random urea 93 mg/dL, and urine protein-to-creatinine ratio 58.3 g/day.

During hospitalization, it was discovered that a year prior the patient underwent CT imaging of the spine for neuropathy and back pain, as well as a CT chest which showed extensive mediastinal adenopathy. The patient was made aware and was given multiple referrals for follow-up. She reports that due to her history of endometrial cancer, she was scared and did not wish to follow up. 

On further workup of her acute lower extremity edema, bilateral lower extremity venous doppler showed no evidence of deep venous thrombosis. A transthoracic echocardiogram showed a normal systolic function with an ejection fraction estimated to be 55%. Renal ultrasound showed a left renal simple cyst measuring 4.1 x 3.3 x 3.7 cm. Torsemide was initiated to an improvement of her edema. 

**Figure 1:**
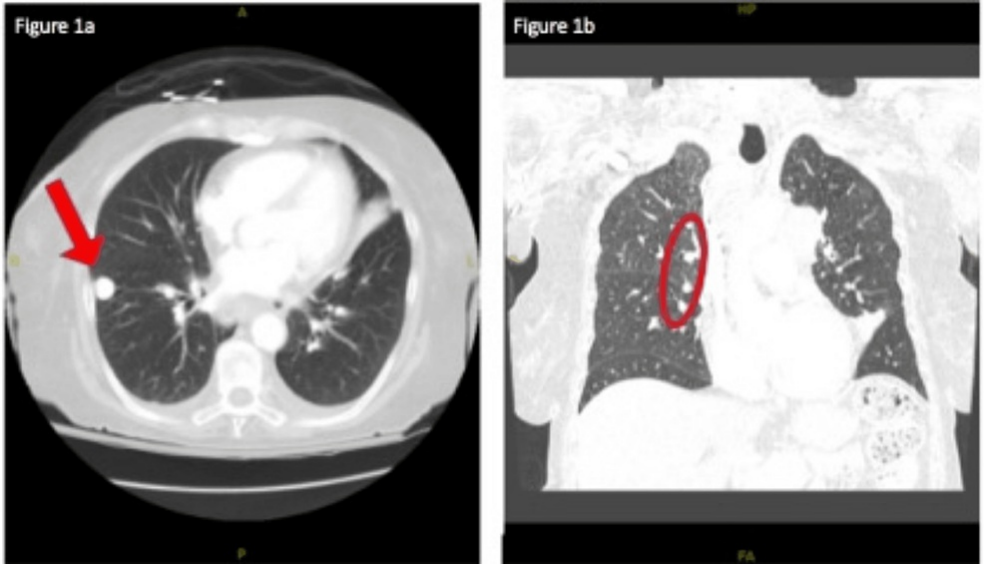
CT chest with contrast. (a) Right-sided coin lesion shown by the red arrow. (b) Mediastinal and left and right hilar adenopathy. Right-sided adenopathy is shown in the red circle.

CT Chest with contrast showed mediastinal and left hilar adenopathy significantly increased from the prior year, with a slight increase in the size of speculation in the right upper lobe measuring 1.5 cm as compared to 1.0 cm in the previous year (Figures 1a, 1b). CT abdomen and pelvis showed indeterminate 1.8 cm right adrenal nodule, unchanged from previous imaging.

Further lab work showed serum protein electrophoresis negative, immunofixation negative, hepatitis Bs antigen negative, hepatitis B core IgM antibody negative, hepatitis C antibody negative, HIV Antigen and antibody negative, ANA positive, anti-double-stranded DNA positive, RNP antibody positive. JO-1 antibody, SS-A antibody, SS-B antibody, Smith antibody, Scl-70 antibody, chromatin antibody, anticentromere antibody, and anti-dorsal root ganglion antibody were negative.

The patient underwent a bronchoscopy for a fine needle aspiration of the right paratracheal lymph node. Biopsy results were positive for synaptophysin and chromogranin indicating metastatic neuroendocrine carcinoma, small cell type. As the patient had significant proteinuria a shared decision was made for a renal biopsy. A CT guided Left renal core biopsy was done resulting in Anti-PLA2R negative, THSD7A positive membranous glomerulopathy and acute tubular injury.

## Discussion

MN is a unique glomerular lesion that can be further classified as primary or secondary MN. Primary MN also known as IMN is the most common cause of renally limited or primary nephrotic syndrome in adults accounting for about 80%. The remaining 20% of cases are classified as secondary MN, which is associated with other systemic diseases or exposures [1-5]. In primary MN, circulating IgG4 autoantibodies to the podocyte membrane antigen PLA2R (anti-PLA2R) have been seen in 70%-80% of individuals with idiopathic MN. However, as in our case, a small percentage of individuals, around 3%-5%, demonstrate THSD7A positivity with or without a concomitant positive anti-PLA2R.

The classic presentation of MN is usually insidious in nature with one-third of patients progressing to end-stage renal disease if not treated early. The diagnosis of MN is based on renal biopsy. Anti-PLA2R was the first antigenic target to be recognized in idiopathic MN since 2009 [3]. THSD7A was described as a second autoantigen involved in IMN. The prevalence of THSD7A has a reported range of 3%-5%. Among individuals with THSD7A-associated MN, studies have shown the development of malignancy within a median follow-up of three months from the time of initial diagnosis [3].

In our case, the patient had a recorded history of mild lower extremity edema two years prior to hospitalization which, along with decreased adherence to medical follow-up, enabled the progression of the nephrotic syndrome. In addition, as MN is insidious in nature, it is likely that this was a prelude to the development of malignancy, which was first noted a year after the onset of lower extremity edema.

As mentioned in the case presentation, it was discovered that the patient developed metastatic neuroendocrine carcinoma, a small cell type. The development of a malignancy in individuals with THSD7A carries a varying incidence of 6%-25% [3]. In addition to the patient's nephrotic syndrome, there were other laboratory findings that may have indicated the development of malignancy and the underlying nephrotic pathology, such as a low anion gap.

Although there is limited data, this case also highlights the possibility of a low anion gap as a useful diagnostic tool. An anion gap value of less than 3 mEq/L is generally considered to be low. The clinical significance of a low anion gap is often unrecognized as there may or may not be an acid-base disturbance in these cases. A low anion gap may be a clinical clue for the diagnosis of life-threatening intoxications, such as lithium or aspirin, or may also be an indication of an occult neoplasm [4]. A primary example of a low anion gap is seen in multiple myeloma, as the monoclonal gammopathy will increase gamma globulins and decrease albumin production. This shift will therefore lead to a decrease in unmeasured anions. With this decrease in anions, there is a compensating physiologic response of elevating serum chloride to maintain homeostasis. In general, other pathologies that may present with a low anion gap due to hypoalbuminemia include cirrhosis, systemic lupus erythematosus, or other classes of nephrotic syndromes. It is therefore important to be aware of these early signs as they may be indicators of underlying pathologies. Although non-specific, a low anion gap with underlying hypoalbuminemia may serve as a clinical clue for the diagnosis of occult neoplasms or other underlying pathologies.

## Conclusions

In summary, this is a case of THSD7A-associated MN which comprises a small percentage of IMN. It may be difficult to discern whether the patient developed MN as a complication of the small cell metastatic neuroendocrine carcinoma, or whether a chronic and insidious THSD7A-associated MN is a risk factor for the development of malignancy. However, due to the symptom onset of lower extremity edema, the latter appears to be the most likely. We shed light on the importance of screening for malignancy in individuals with this subtype of MN. We also bring forward the use of a low anion gap (anion gap value < 3 mEq/L) as a useful diagnostic tool for the diagnosis of life-threatening intoxications, occult neoplasms, and other possible pathologies.
